# Safety and feasibility of home-based transcranial alternating current stimulation in youths with 22q11.2 deletion syndrome

**DOI:** 10.3389/fnins.2024.1453839

**Published:** 2024-10-24

**Authors:** Caren Latrèche, Valentina Mancini, Nova McGinn, Vincent Rochas, Victor Férat, Silas Forrer, Maude Schneider, Stephan Eliez

**Affiliations:** ^1^Developmental Imaging and Psychopathology Lab, Department of Psychiatry, University of Geneva School of Medicine, Geneva, Switzerland; ^2^Functional Brain Mapping Laboratory, Department of Basic Neurosciences, University of Geneva, Geneva, Switzerland; ^3^Human Neuroscience Platform, Fondation Campus Biotech Geneva, Geneva, Switzerland; ^4^Medical Image Processing Laboratory, Neuro-X Institute, École Polytechnique Fédérale de Lausanne, Lausanne, Switzerland; ^5^Psychology Unit for Developmental and Intellectual Disabilities, Faculty of Psychology and Educational Sciences, University of Geneva, Geneva, Switzerland; ^6^Department of Genetic Medicine and Development, University of Geneva School of Medicine, Geneva, Switzerland

**Keywords:** 22q11 deletion syndrome, neurodevelopmental disorders, non-invasive brain stimulation, transcranial alternating current stimulation (tACS), safety and feasibility

## Abstract

Neurodevelopmental disorders such as attention deficit and/or hyperactivity disorder (ADHD) and schizophrenia are characterized by core impairment in executive functions (EF). Despite the development of various behavioral interventions to enhance EF, the evidence is still scarce. Alternatively, non-invasive brain stimulation tools such as transcranial alternating current stimulation (tACS) has emerged as a potential strategy to alleviate cognitive deficits. Previous studies have demonstrated the safety, feasibility, and efficacy of one single tACS session in different clinical populations. However, the effects of tACS appear limited and need to be sustained to be considered an effective cognitive neurorehabilitation tool. Recent studies have used home-based, repeated tACS sessions in individuals with neurodegenerative diseases. To our knowledge, the safety and feasibility of such an intensive protocol remains to be tested in a younger population with neurodevelopmental disorders. Using a randomized double-blind sham-controlled design, we administered home-based, repeated tACS sessions to seven individuals aged 14–25 with 22q11.2 deletion syndrome (22q11.2DS), which confers an increased risk for neurodevelopmental disorders. We aimed to assess the safety, tolerability, and feasibility of tACS. Findings from this ongoing clinical trial revealed a favorable safety profile, with frequent yet transient and mainly mild adverse effects. The intervention proved to be feasible, shown by very high adherence rates and positive user experiences. Future studies should therefore investigate whether prolonged exposure to tACS can lead to long-lasting cognitive outcomes.

**Clinical trial registration:**

ClinicalTrials.gov, identifier NCT05664412.

## 1 Introduction

Deficits in executive functions (EF) are pervasive and shared by several neurodevelopmental disorders such as autism spectrum disorder (ASD), attention-deficit/hyperactivity disorder (ADHD), and schizophrenia (Dajani et al., 2016). In these disorders, impaired EF have been associated with poorer clinical and functional outcomes (Biederman et al., 2006; González-Ortega et al., 2013; Wong et al., 2023). To improve EF in young individuals with neurodevelopmental disorders, numerous behavioral interventions were developed, particularly targeting working memory (WM; Al-Saad et al., 2021; Bharadwaj et al., 2022). While some studies yielded significant cognitive improvements following WM training, effect sizes are overall small, and transfer effects, limited (Bombonato et al., 2024). Alternative interventions are thus needed to effectively enhance WM in neurodevelopmental disorders.

Over the past decade, transcranial alternating current stimulation (tACS) has emerged as a promising therapeutic strategy to enhance cognitive functions by modulating underlying oscillatory activity with a low-intensity sinusoidal electrical current (Hanslmayr et al., 2019). Prior research has demonstrated that aberrant neuronal oscillations underlie WM impairments in several conditions such as schizophrenia and neurological disorders (Berger et al., 2016; Assenza et al., 2017; Abubaker et al., 2021). In particular, the phase-amplitude coupling (PAC) between theta and gamma oscillations is recognized as neurophysiological signature of WM (Lisman and Buzsáki, 2008; Fell and Axmacher, 2011; Papaioannou et al., 2022). Interestingly, alterations in theta-gamma PAC are a recurrent finding in neurodevelopmental, neurodegenerative, and psychiatric populations (Yakubov et al., 2022). Studies using tACS revealed an excellent safety profile (Antal et al., 2017; Matsumoto and Ugawa, 2017). Other favorable aspects of tACS include its portability, tolerability, and low cost (Bland and Sale, 2019). In addition, a recent meta-analysis, highlighted the effectiveness of tACS in improving WM in healthy, aging, and psychiatric populations (Grover et al., 2023). Several studies have used various tACS protocols to attenuate WM deficits (Al Qasem et al., 2022), yet only few specifically targeted theta-gamma PAC (Reinhart and Nguyen, 2019).

Here we focus on 22q11.2 deletion syndrome (22q11.2DS), a neurogenetic disorder characterized by an increased risk for neurodevelopmental and psychiatric disorders, and by mild intellectual disability to borderline intellectual functioning (McDonald-McGinn et al., 2015; Fiksinski et al., 2021; Schneider et al., 2014). Moreover, individuals with 22q11.2DS show an atypical development in EF including WM (Jhawar et al., 2021; Maeder et al., 2016, 2021). Furthermore, they exhibit aberrant theta- and gamma-band oscillations and theta-gamma PAC (Mancini et al., 2022a; Mancini et al., 2022b). To alleviate WM deficits in 22q11.2DS, a recent tACS study was conducted in 34 youths using a double-blind sham-controlled design (Latrèche et al., 2024). The stimulation protocol consisted in one session of bifocal theta-tuned tACS, targeting prefrontal and temporal brain areas. Personalized parameters were used to maximize the effectiveness of tACS (Grover et al., 2021). The intervention proved to be well-tolerated, as the reported mild adverse effects (i.e., tingling, sleepiness) were expected (Antal et al., 2017). Moreover, a significant increase in WM performance after one 20-min tACS session was revealed in comparison to sham tACS (8.9% increase). Yet, before considering tACS as a potential cognitive rehabilitation tool, studies must investigate whether repeated exposure to tACS may consistently yield long-lasting cognitive outcomes.

Studies using repeated tACS sessions have only been seldom conducted so far, especially in clinical populations. Numerous practical and methodological constraints come with designing a study with multiple stimulation sessions in individuals with cognitive impairment. Nevertheless, recent advances in brain stimulation technology allowed to make tACS more accessible and affordable, by creating home-based study protocols that allow for real-time remote supervision. Such home-based, remotely-monitored and caregiver-administered protocols have mainly been conducted with older adults diagnosed with neurodegenerative diseases (Bréchet et al., 2021; Cappon et al., 2023). Pilot findings support the tolerability and the efficacy of tACS on memory functions after over 70 sessions. However, implementing such intensive protocols in a younger population with neurodevelopmental disorders poses an even greater challenge. Indeed, the educational and academic demands often combined with additional therapeutic interventions can result in overwhelming schedules for both affected individuals and caregivers. Consequently, to our knowledge, no studies have been yet carried out in youths with neurodevelopmental and psychiatric disorders.

In this preliminary study, we therefore aimed to first assess the safety and tolerability of home-based, repeated tACS sessions in youths with 22q11.2DS. Secondly, we aimed to assess the feasibility and adherence to this intensive tACS protocol. Given our previous findings (Latrèche et al., 2024), we hypothesized that tACS would be safe, well-tolerated, and feasible in our sample, with mainly mild and transient adverse effects and high compliance rates.

## 2 Method

### 2.1 Participants

Participants were recruited from the Swiss 22q11.2DS longitudinal cohort and were enrolled in the present study from October 17th 2023 to May 27th 2024. The sample included seven participants (3 females), with a mean age of 19.85 years (SD = 4.42, range = 14–25). Participants’ baseline demographic and clinical characteristics are presented in Table 1 and Supplementary Table S1. Written informed consent based on protocols approved by the Swiss Ethical Committee of Geneva (CCER) and the Swiss Agency for Therapeutic Products (Swissmedic) was obtained from participants or parents (for participants younger than 18 years).

**Table 1:** Participants’ demographic and clinical characteristics, and personalized stimulation parameters.

Case	Age	Sex	Frequency (Hz)	Intensity (mA)
1	25	M	4	1.55
2	25	M	4.8	1.55
3	20	F	4	1.5
4	14	F	4	1.05
5	17	F	5.4	1.05
6	17	M	4	1.55
7	16	M	4.2	1.7

Inclusion criteria were the following:

1. 22q11.2DS diagnosis confirmed by quantitative fluorescent polymerase chain reaction (PCR)
2. Minimum age of 12 years or maximum age of 25 years and 11 months
3. Sufficient verbal and comprehension capacity to understand and follow task instructions

Exclusion criteria were as follows:

1. History of epilepsy
2. History of deep brain stimulation
3. History of traumatic brain injury
4. Metal implants in the head area, cochlear implants or other devices implanted in the head area

### 2.2 Study design

**Figure 1:**
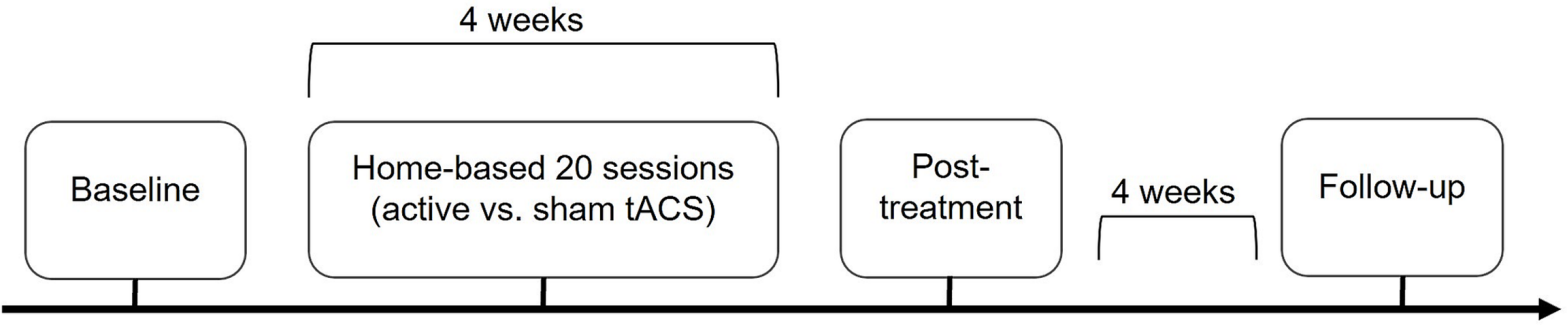
Study design.

In this randomized double-blind sham-controlled clinical trial, we have used the Starstim®-Home Kit, a home-based tACS device developed by Neuroelectrics Corp.^1^ The participants completed three assessments (i.e., baseline, post-treatment, follow-up). After the baseline visit, they completed a 4-week treatment phase, followed by a post-treatment visit and a 4-week follow-up visit (Figure 1). The baseline assessment comprised an MRI scan at rest and a high-density (HD-)EEG recording using a 256-electrode HydroCel cap (Electrical Geodesics, Inc.) during a visual WM task. Participants and their caregiver(s) were given instructions on the tACS device and were trained to use it in a mock session during the baseline assessment, with the help of trained staff.

The treatment phase entailed 20 home-based and remotely supervised active or sham tACS sessions, including five 20-min sessions per week for a duration of 4 weeks. The sessions were all scheduled at the same time of day (morning/afternoon/end of day) depending on the availability of the participant and their caregiver(s). Real-time monitoring was performed remotely by the staff through video-conferencing using Zoom.^2^ The staff were able to guide and correct participants and caregivers during set-up, thus ensuring optimal treatment fidelity. After the setup, participants started the stimulation session and completed a behavioral task displayed via screen sharing. The behavioral task consisted in two parallel versions of a 8-min visual WM task, as used in our previous tACS study (Latrèche et al., 2024). Participants were required to respond orally to each trial, their answers being recorded simultaneously by the experimenter. At the end of the session, the presence and intensity of adverse effects were assessed using a systematic questionnaire (Brunoni et al., 2011).

To assess any potential short- and long-term adverse effects of tACS, post-treatment and follow-up assessments, respectively, took place on average 3 days (SD = 0.58) and 28 days (SD = 1.25) after the last home-based tACS session.

### 2.3 tACS protocol and administration

**Figure 2:**
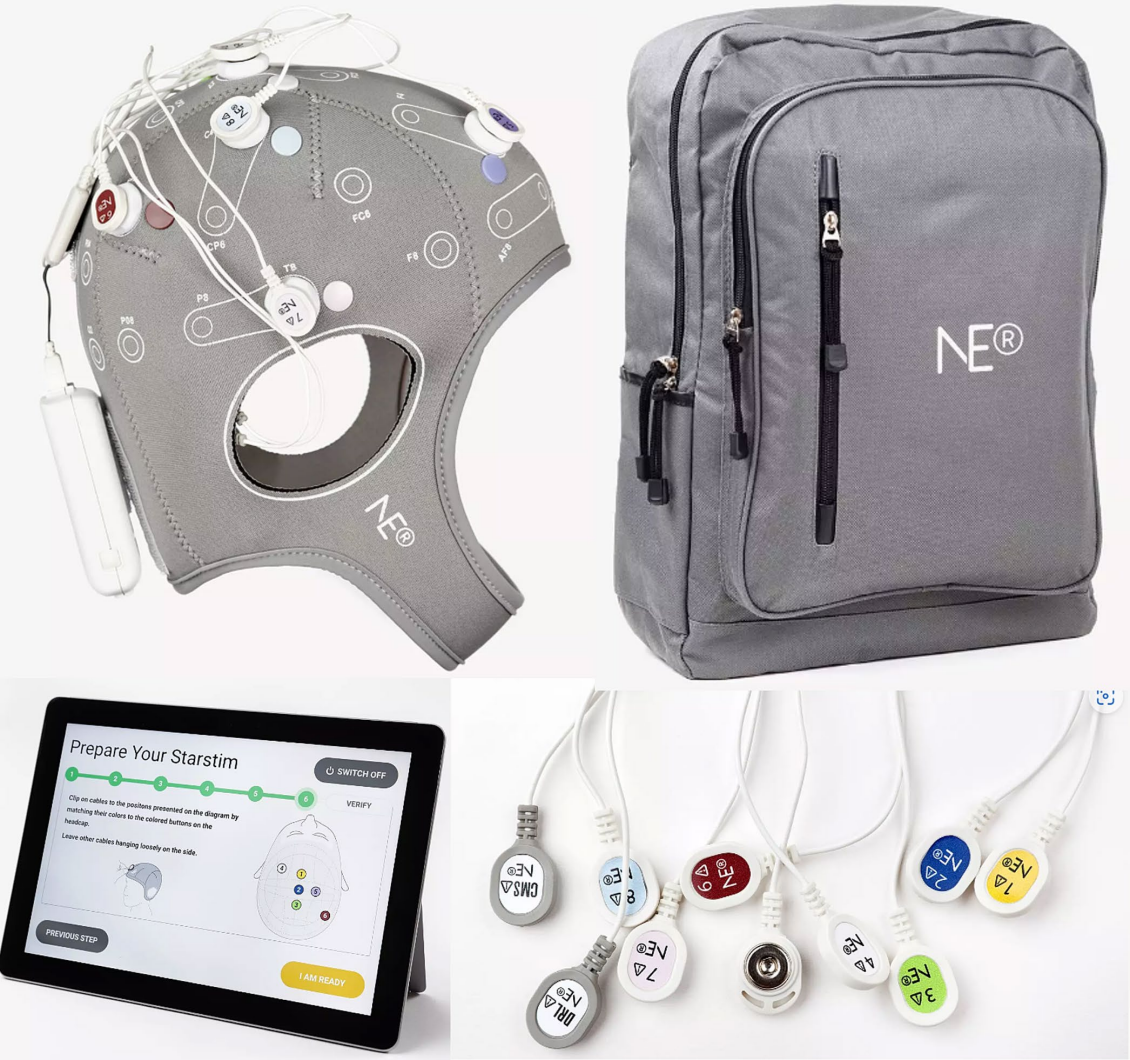
Neuroelectrics Starstim® Home Kit including the headcap, tablet, and color- and number-coded electrodes.

The Starstim® Home Kit was used to deliver tACS in our study (Figure 2). We chose a bifocal montage using 5 Pistim (Ag/AgCL) electrodes with a *π* cm^2^ circular contact area, with two targeting the left PFC and three targeting the left temporal cortex, respectively. These electrodes were inserted in a Neoprene cap which includes 39 defined positions based on the international 10–20 EEG system. The five selected positions were AF4, AF7, Fpz for the frontal target and T7 and P7 for the temporal target. Color- and number-coded electrodes were fixed on the five selected positions using conductive gel between each electrode and the scalp to reduce impedance and convey the stimulation current. Subsequently, the electrodes were connected by a cable, which was plugged into the Necbox. The Necbox was placed on the back of the Neoprene cap and wirelessly paired with a Home Tablet. Step-by-step instructions on preparation and maintenance were provided in the Home Tablet.

Our tACS protocol was based on stimulation parameters tailored to the individual brain, to account for the functional and anatomical variability between participants. Such parameters were derived from baseline HD-EEG and structural MRI data to, respectively, compute the frequency and model the current intensity of tACS, as in previous studies (Reinhart and Nguyen, 2019; Latrèche et al., 2024). First, the individual frequency of stimulation within the theta range (4–8 Hz) was estimated based on a HD-EEG recording during a visual WM task. The theta frequency with the maximal phase-amplitude coupling (PAC) between the theta phase in prefrontal and the amplitude of gamma in temporal regions was selected for each participant. This analysis was based on a pipeline already applied to previous data (Mancini et al., 2022b; Latrèche et al., 2024) and described in detail in the Supplementary material. Second, the individual electrode intensity values were computed based on a modeling from T1- and T2-weighted scans acquired with a 3 T Siemens Prisma scanner at the Human Neuroscience Platform, Fondation Campus Biotech Geneva (see the Supplementary material for detailed information on MRI parameters). Both scans were used to perform electrical current flow modeling with SimNIBS v.3 (Thielscher et al., 2015). The intensity value for each electrode was limited to a maximum of 1 mA (2 mA for the five electrodes), therefore substantially below injury threshold levels (Bikson et al., 2016). These values were adjusted to produce a focal electrical field of 0.3 V/m in the two targeted brain regions (i.e., left prefrontal and temporal cortex). The active tACS session started with a 20 s ramp up, followed by a 20-min session sustained at the individualized stimulation frequency and intensity, and ended with a 20 s ramp down. The sham tACS consisted in a 20 s ramp up-and-down at the beginning and end of the session. The session duration was consistent with previous studies (Weickert et al., 2019; Latrèche et al., 2024). Personalized frequency and intensity values for the seven participants are shown in Table 1.

### 2.4 Randomization

Participants were randomized to receive either active or sham (1:1 allocation ratio). A staff member (SF) external to the current study performed a random allocation sequence comprising alternating blocks of 2, 4, 6, and 8 participants. The same staff member allocated treatment (sham or active tACS) in the NIC2 Neuroelectrics software and exported the stimulation protocol. The stimulation protocol was uploaded by the study coordinator (CL) on the Neuroelectrics portal through the blinded mode.^3^ The 20 sessions were remotely scheduled. The portal displayed whether the session was executed, missed, or aborted. If a session was incomplete, details were provided about the incident that occurred, and the total time completed. Unblinding was performed after the follow-up visit of each participant.

### 2.5 Material

#### 2.5.1 Safety and tolerability

Safety and tolerability were assessed after each active and sham tACS session and at the post-treatment and follow-up visits. To assess side effects, we used a systematic questionnaire comprising potential adverse events commonly related to non-invasive brain stimulation (Brunoni et al., 2011). Participants were asked to report the presence and intensity of side effects (ranging from 1 = absent to 4 = severe), as well as their relation to tACS (from 1 = no link to 5 = certain link).

#### 2.5.2 Feasibility

Adherence to home-based tACS was assessed similarly to previous studies (e.g., Cappon et al., 2022). We computed the ratio of completed tACS sessions to the total number of sessions required by our protocol (20 sessions). In addition, participants and caregivers were asked to fill a user experience questionnaire (UEQ^4^; Schrepp et al., 2014) after the end of the active and sham tACS sessions, regarding their use of the Starstim®-Home Kit. The 26 items of the UEQ are summed in the six following scales: attractiveness, efficiency, perspicuity, dependability, novelty, and stimulation. Each scale has a mean value, where values between −0.8 and 0.8 represent neutral evaluation, values >0.8 represent a positive evaluation and <0.8 a negative one.

## 3 Results

### 3.1 Safety and tolerability

**Figure 3:**
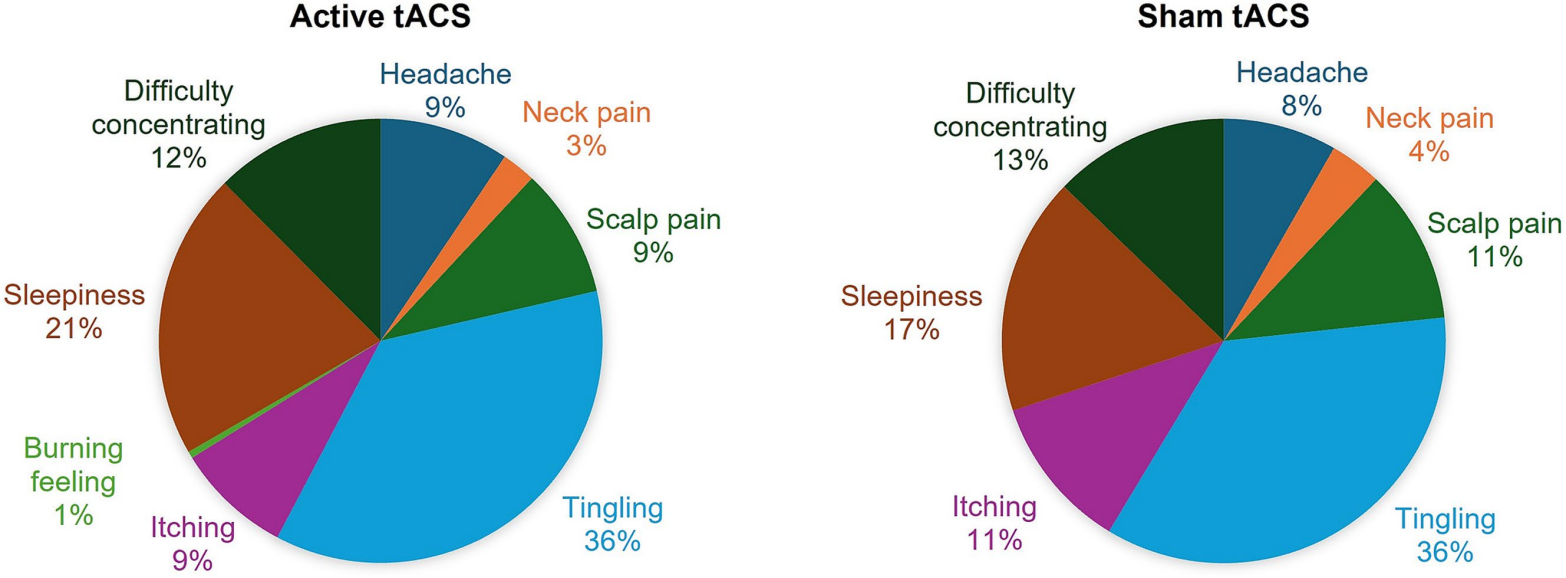
Distribution of adverse effects in active and sham tACS groups.

Figure 3 and Supplementary Table S2 show the reported adverse events during the 20 home-based tACS sessions in the active and sham tACS groups, respectively. No sessions were interrupted due to discomfort, and no participants were withdrawn from the clinical trial due to serious adverse events. Participants from both groups reported frequent and transient adverse events. Across all completed sessions, the intensity was not significantly different between active and sham tACS. Participants reported mostly mild [86.1% vs. 80.5%, for active vs. sham tACS respectively, *χ^2^*(1,7) = 0.907, *p* = 0.341] compared to moderate [13.9% vs. 18%, *χ^2^*(1,7) = 0.595, *p* = 0.440] and severe [0% vs. 1.5%, *χ^2^*(1,7) = 2.020, *p* = 0.155] adverse effects throughout the treatment phase.

Tingling was assessed as probably related to stimulation in both active and sham tACS groups. Additionally, it was the most frequent adverse effect in both groups, across all active (93%) and sham (79%) tACS sessions, with significantly more reports in the former group [*χ^2^*(1,7) = 8.140, adjusted *p* = 0.032]. The occurrence of other adverse effects did not differ between groups, as shown in Supplementary Table S2. Participants commonly reported other adverse effects with a possible link to stimulation, such as itching (21% vs. 25%, for active vs. sham tACS respectively), headache (24% vs. 20%), and scalp pain (22% vs. 25%). Other frequent side effects were reported but only with a remote link to stimulation, such as sleepiness (54 and 39%) and concentration problems (32 and 27%). Burning feeling and neck pain were only very rarely reported in active and sham tACS groups (1% vs. 0 and 6% vs. 9%, respectively), whereas skin redness was not reported in the sample.

No adverse events at the post-treatment assessment were reported by participants from the sham tACS group. In the active tACS group, 2/3 participants reported mild and moderate headache remotely linked to tACS. No adverse events were reported at the 4-week follow-up visit in either group.

### 3.2 Feasibility

A very high adherence to the tACS protocol was observed in our seven participants, with a percentage of 96.43% sessions fully completed (135 out of the 140 scheduled sessions). Of the seven participants, four completed all 20 sessions. One participant from the active tACS group and one from the sham tACS group each experienced one incomplete session, due to an auto-abortion on high impedances (68% completed) and on Necbox disconnection (85% completed), respectively (see Supplementary Table S3). In total, three sessions (2.14%) were missed due to technical problems in relation to the device. Two sessions were missed by one participant from the active tACS group, and one session was missed by a participant from the sham tACS group.

Regarding user experience with the Starstim®-Home Kit, results from the UEQ are available in Supplementary Table S4. Participants and caregivers positively evaluated the device in terms of perspicuity (i.e., ease of use), efficiency (i.e., solving tasks without unnecessary effort), dependability (i.e., sense of control), and novelty (i.e., innovation and creativity of the product). Neutral evaluations were given for the attractiveness (i.e., overall impression) and stimulation (i.e., excitement and motivation to use the product) dimensions.

## 4 Discussion

This preliminary study investigated the safety and feasibility of 20 home-based tACS sessions in a double-blind randomized clinical trial with individuals with 22q11.2DS. To our knowledge, this is the first study using home-based tACS with a young population with a neurogenetic condition and cognitive deficits characterized by high risk for developing psychiatric disorders.

Safety and tolerability were repeatedly assessed over the treatment phase, as well as at the post-treatment and 4-week follow-up visits. Participants did not report any serious adverse events and did not abort any sessions due to intolerable pain or discomfort. Nevertheless, they reported frequent adverse effects of predominantly mild intensity. These results were expected, as we followed a conventional stimulation protocol with a total intensity of ≤2 mA and 20 min per day, in line with safety guidelines (< 4 mA with up to 60 min per day, Antal et al., 2017). Our findings are also consistent with previous research showing minor adverse effects following non-invasive brain stimulation in healthy and clinical populations of different ages (Brunoni et al., 2011; Krishnan et al., 2015; Matsumoto and Ugawa, 2017). In particular, children and adolescents were showed to mainly report a tingling sensation following transcranial current stimulation (Krishnan et al., 2015), which is again in line with both our present and previous studies (Latrèche et al., 2024). Besides, at the post-assessment visit (i.e., 3 days after the last session), no adverse effects were reported as directly linked to tACS. Only headache with a remote link to tACS was reported by half of the active tACS group. Moreover, participants did not report any adverse effects at the 4-week follow-up visit, demonstrating the transient nature of such effects (Antal et al., 2017). Therefore, we provide encouraging evidence for the safety and tolerability of repeated exposure to tACS in youths with 22q11.2DS.

Participants showed a very high level of adherence to our tACS protocol, with 135/140 sessions completed. Reasons for incomplete or missed sessions were directly linked to technical issues with the Starstim®-Home Kit (e.g., auto-abortion due to Necbox disconnection or to high impedances, broken electrode). Such high adherence rates were certainly achieved by the combination of both direct and remote supervision of tACS sessions. First, the direct presence of caregivers was essential for practical aspects (i.e., tACS set-up). Moreover, caregivers showed high levels of engagement, as demonstrated by their commitment to this intense protocol that required numerous adjustments in their daily lives during the 4 weeks of stimulation sessions. Therefore, their support was a key factor in optimizing participant compliance and the feasibility of the protocol. Second, the real-time remote supervision by trained staff via videoconferencing allowed to guide caregivers and ensure a correct set-up. In addition, daily social interactions with researchers may have represented a source of motivation and engagement for participants. The presence of both participants and caregivers is therefore ideal for the successful conduct of the stimulation sessions. Yet, such an intensive protocol can be challenging to fit in the schedule of individuals with 22q11.2DS given both educational and professional demands, as well as the multidisciplinary care that is often required (Boot et al., 2023; Óskarsdóttir et al., 2023). Consequently, most sessions took place in the late afternoons or early evenings, which may have exacerbated reports of mild sleepiness and difficulty concentrating in our sample. Indeed, fatigue and attention difficulties are part of the clinical phenotype in 22q11.2DS (Vergaelen et al., 2017; Reich et al., 2023; Schneider et al., 2014).

We examined the participants and caregivers’ experience with the use of the Starstim®-Home Kit. Participants and caregivers positively evaluated the device on most dimensions of the UEQ. They particularly appreciated its efficiency, reliability and innovation. More neutral evaluations were given for the attractiveness and excitation/motivation dimensions, likely given the length of the treatment phase which may have tempered the initial enthusiasm.

This work comes with several limitations. First, these preliminary results are part of an ongoing clinical trial, which explains the small sample size. Second, our sample is heterogeneous in terms of psychiatric diagnoses and psychotropic medication use. Nevertheless, psychotropic treatments were introduced prior to the start of the study and were stable over its course. Third, while one tACS session significantly enhances WM in 22q11.2DS (Latrèche et al., 2024), we have no evidence yet of the efficacy of multiple tACS sessions. Future studies should investigate both short- and long-term effects of home-based tACS on WM, along with its potential transfer effects on other cognitive functions.

## 5 Conclusion

This randomized double-blind sham-controlled clinical trial offers preliminary evidence for the safety, tolerability, and feasibility of home-based, repeated tACS in youths with 22q11.2DS. Using a personalized tACS protocol in accordance with safety guidelines and a real-time remote monitoring were essential in ensuring a favorable safety profile and high rates of adherence to the study.

## Data Availability

The raw data supporting the conclusions of this article will be made available by the authors, without undue reservation.

## Footnotes

^1^ https://www.neuroelectrics.com/solutions/starstimhome

^2^ https://zoom.us/

^3^ https://portal.neuroelectrics.com

^4^ https://www.ueq-online.org/
