# Supplementary material for: Safety and feasibility of home-based transcranial alternating current stimulation in youths with 22q11.2 deletion syndrome
Source: Front Neurosci. 2024 Oct 24;18:1453839. doi: 10.3389/fnins.2024.1453839 (PMC11541232; doi:10.3389/fnins.2024.1453839)
Supplement: Supplementary file 1 [file Data_Sheet_1.DOCX]

Supplementary Material

# Supplementary Methods

**MRI acquisition**

T1- and T2-weighted scans were obtained using a 3T Siemens Prisma scanner at the Human Neuroscience Platform, Fondation Campus Biotech Geneva. The T1-weighted MPRAGE sequence parameters for structural image acquisition were TR = 2500 ms, TE = 3 ms, flip angle = 8°, acquisition matrix = 256 × 256, field of view = 23.5 cm, voxel size = 0.9 × 0.9 × 1.1 mm and 192 slices. The T2-weighted sequence parameters for structural image acquisition were TR = 3000 ms, TE = 409 ms, field of view = 25.6 cm, voxel size = 0.8× 0.8 × 0.8 mm and 208 slices.

**EEG acquisition and preprocessing**

EEG data were continuously recorded at a sampling rate of 1000 Hz using a 256-electrode HydroCel cap (EGI-Philips Healthcare) referenced to the vertex (Cz) during the working memory task at the Human Neuroscience Platform, Fondation Campus Biotech Geneva. The impedance was kept below 30 kΩ for all electrodes and below 10 kΩ for the reference and ground electrodes.

The free academic software Cartool (<https://sites.google.com/site/cartoolcommunity/home>; Michel and Brunet, 2019) was used to perform the pre-processing steps, as described in previous studies (Mancini et al., 2022a, 2022b). Specifically, the number of electrodes was reduced from 256 to 204 by removing the noisy electrode signals from the cheeks and neck. The data were band-pass filtered between 1 and 140 Hz using non-causal Butterworth filters, with additional notch filters applied at 50, 100 and 150 Hz. The periods of artifacts (e.g., muscle contraction) were marked manually and excluded from further analysis. Noisy channels were identified by through visual inspection and subsequently excluded. Independent Component Analysis (ICA) was used to remove eye-movements (eye blinks and saccades) and ECG artefacts components using a Matlab script based on the EEGlab runica function (<https://sccn.ucsd.edu/eeglab/>; Makeig et al., 1997). The identified noisy channels were interpolated using a 3D spline interpolation (Perrin et al., 1989). Finally, data were recalculated to the common average reference.

**Phase-amplitude coupling estimation**

The pre-processed EEG data were filtered into the theta (4-8 Hz) and gamma (25-35 Hz) frequency bands of interest, and then segmented into epochs from – 0.5 to +1.5 seconds. The clean concatenated EEGs respectively filtered for theta and gamma frequency bands were transformed to singular value decomposition (SVD) of the ROIs of interest (left PFC and left temporal cortex) using an in-house toolbox (PyCartool; Mancini et al., 2022b) with individual IS model and parcellation atlas. The real SVD signals of each ROIs obtained for each frequency band were computed through Hilbert transform. The instantaneous angles of the SVD for theta frequency band were sorted from complex values. Then, the gamma amplitude envelope of the ROIs of interest was obtained from the complex values of the SVD for the gamma frequency band. Coupling between ROIs was estimated with the modulation index (MI), as described by Tort et al. (2010) taking into account the 0 to 0.5 sec periods after the onset of stimulus. In detail, a composite time series was computed as the averaged amplitude of gamma in the temporal cortex at each binned phase of theta rhythm in the PFC. The amplitude distribution was obtained normalizing the mean amplitude by dividing each bin value over the sum of the bins. MI was then defined as the Kullback-Leibler distance of the observed amplitude distribution (P) from the uniform distribution (U) divided by the logarithm of N (i.e., the maximum possible entropy value). Finally, we calculated the theta frequency corresponding to the highest value of PAC, i.e., to the highest value of the MI.

# Supplementary Tables

**Supplementary Table 1.** Psychiatric diagnoses and psychotropic medication use

|  |  | Participants, n(%) |
| --- | --- | --- |
| N |  | 7 |
| Psychiatric diagnosis (%) |  |  |
| Total |  | 5 (71.43) |
| Categories |  |  |
|  | Attention deficit disorder | 3 (42.86) |
|  | Generalized anxiety disorder | 1 (14.29) |
|  | Mood disorder | 1 (14.29) |
|  | Psychosis spectrum disorder | 1 (14.29) |
|  | Social phobia | 2 (28.57) |
|  |  |  |
| Medication (%) |  |  |
| Total |  | 7 (100) |
| Categories | Psychostimulants | 5 (71.43) |
|  | Antidepressants | 5 (71.43) |

**Supplementary Table 2.** Detailed distribution of adverse effects in active and sham tACS groups

|  | Post-active tACS | | | Post-sham tACS | | | Comparison  active vs. sham tACS | | |
| --- | --- | --- | --- | --- | --- | --- | --- | --- | --- |
|  | Mild, *n*(%) | Moderate, *n*(%) | Severe, *n*(%) | Mild, *n*(%) | Moderate, *n*(%) | Severe, *n*(%) | Pearson’s  Chi square | *p* value | adjusted *p* value |
| Headache | 13 (16.7) | 6 (7.5) | 0 | 8 (15) | 3 (5) | 0 | 0.466 | 0.495 | 0.614 |
| Neck pain | 5 (6.25) | 0 | 0 | 4 (7.5) | 1 (1.7) | 0 | 0.649 | 0.421 | 0.614 |
| Scalp pain | 18 (21.1) | 1 (1.3) | 0 | 11 (19.6) | 4 (5) | 0 | 0.250 | 0.617 | 0.617 |
| Tingling | 66 (84.2) | 7 (8.8) | 0 | 39 (65) | 8 (14.2) | 0 | 8.140 | 0.004 | **0.032** |
| Itching | 17 (21.25) | 0 | 0 | 14 (22.9) | 1 (1.7) | 0 | 0.452 | 0.502 | 0.614 |
| Burning feeling | 1 (1.3) | 0 | 0 | 0 | 0 | 0 | 1.005 | 0.316 | 0.614 |
| Skin redness | 0 | 0 | 0 | 0 | 0 | 0 | - | - | - |
| Sleepiness | 28 (35.8) | 14 (17.9) | 0 | 15 (25) | 6 (10.8) | 2 (3.3) | 4.522 | 0.034 | 0.136 |
| Difficulty concentrating | 25 (31.7) | 0 | 0 | 16 (26.7) | 1 (1.7) | 0 | 0.381 | 0.537 | 0.614 |

**Supplementary Table 3.** Number of completed, incomplete, and missed sessions across the 20 active vs. sham tACS sessions per participant.

|  |  | Across the 20 active or sham tACS sessions | | |
| --- | --- | --- | --- | --- |
|  | Case | Completed | Incomplete | Missed |
| Active  tACS  group | 1 | 20 | 0 | 0 |
|  | 2 | 20 | 0 | 0 |
|  | 3 | 17 | 1 | 2 |
|  | 4 | 20 | 0 | 0 |
| Sham tACS  group | 5 | 20 | 0 | 0 |
|  | 6 | 19 | 1 | 0 |
|  | 7 | 19 | 0 | 1 |

**Supplementary Table 4.** Results of the six scales from the user experience questionnaire (UEQ). Each scale has a mean value, where values between -0.8 and 0.8 represent neutral evaluation, values > 0.8 represent a positive evaluation, and <0.8 a negative one.

| UEQ Scales | Mean (SD) |
| --- | --- |
| Attractiveness | 0.595 (2.30) |
| Perspicuity | 2.107 (1.35) |
| Efficiency | 1.214 (1.15) |
| Dependability | 1.321 (0.41) |
| Stimulation | 0.321 (2.45) |
| Novelty | 1.250 (1.44) |
